# *S. mutans* induces developmental and morphological defects in *C. elegans* despite prior *L. casei* exposure

**DOI:** 10.17912/micropub.biology.001846

**Published:** 2025-12-15

**Authors:** Ana M. Cedeno Escobar, Katherine M. Walstrom

**Affiliations:** 1 Division of Natural Sciences, New College of Florida, Sarasota, Florida, United States

## Abstract

Imbalances between pathogenic and commensal bacteria cause microbial dysbiosis that disrupts host development. *Streptococcus mutans '* virulence factors promote human oral cell colonization, leading to dysbiosis if unchecked. When grown with the probiotic *Lacticaseibacillus casei* , a competitive relationship ensues. Using *Caenorhabditis elegans* , two assays quantified the effects of these bacteria on egg production and worm growth. Worms exposed to *S. mutans* exhibited fewer offspring, egg defects, delayed development, and twisted pharynx and dumpy phenotypes. *L. casei* did not prevent these effects.

**Figure 1. Developmental effects of bacterial treatments on C. elegans egg production, larval growth, and morphology:**
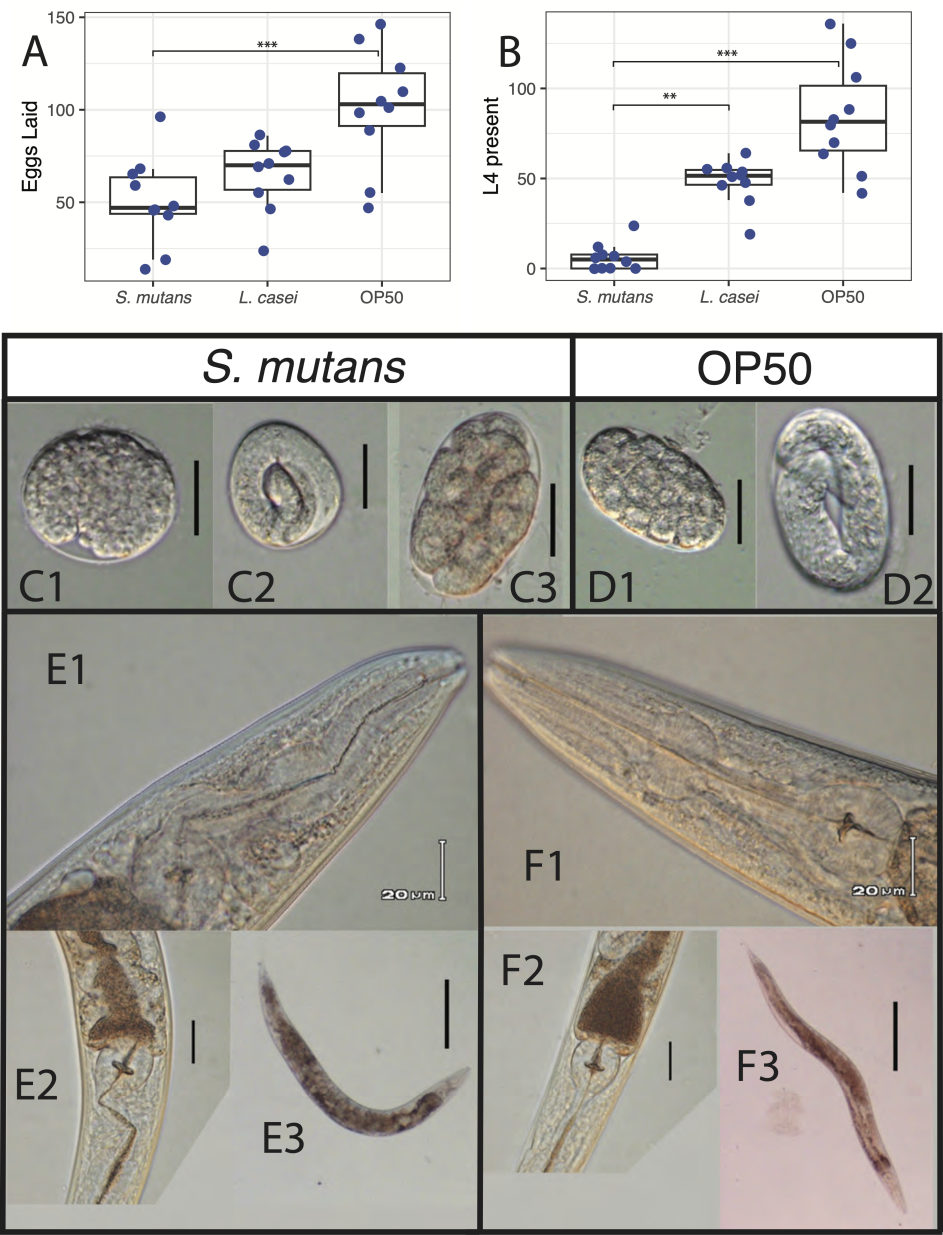
**(A)** Number of eggs recorded for each bacterial treatment in Assay 1. A Kruskal-Wallis test ( *H* = 12.47, *df* = 2, p = 0.001) indicated a significant difference between *S. mutans* and OP50 treatments (p = 0.001). **(B)** Number of L4 recorded across bacterial treatments in Assay 1. The Kruskal-Wallis test ( *H* = 22.47, *df* = 2) showed significant differences between *S. mutans* and OP50 (p < 0.001), and between *S. mutans* and *L. casei* (p = 0.013). **(C)** DIC images of eggs from *S. mutans* treatments. C1 and C2 show round eggs at different developmental stages. C3 shows an example of an oval egg. **(D)** DIC images of control eggs from OP50 treatments. D1 and D2 show a normal oval shape and embryo development. **(E)** Images of adult worms treated with *S. mutans* . E1 and E2 show twisted pharynx phenotypes. E3 shows a dumpy worm with reduced body length. **(F)** Images of adult worms from the OP50 control. F1 and F2 display normal pharynx morphology, and F3 shows normal body size and shape. The size bars are 20 μm long in C, D, E1 and F1, 40 μm in E2 and F2, and 200 μm in E3 and F3. *** indicates p ≤ 0.001 and ** indicates p ≤ 0.05.

## Description

The term microbial dysbiosis describes an imbalance of the microbiome, and this imbalance can facilitate cancer progression (Whisner and Aktipis, 2019). *Streptococcus mutans* ( *S. mutans* ), a cariogenic bacterium, utilizes its capacity for adherence, acid production, and acid tolerance to collaborate with other bacteria to invade host cells; thereby promoting oral squamous cell carcinoma and other extra-oral diseases (Han and Wang, 2013, Tsai et al., 2022). Its pathogenic potential is attributed to virulence factors, described as molecules that aid microorganisms during colonization or when damaging the host's cells (Sharma et al., 2017). In contrast, *Lacticaseibacillus casei* ( *L. casei* ) exhibits probiotic properties shown to assist in restoring microbial homeostasis for the intestinal flora (Fijan, 2014, Chang et al., 2018). The protective and restorative effects of *L. casei* on the gut microbiota have been recently demonstrated in *C. elegans* and human intestinal cells (Bae et al., 2025, Kang et al., 2025, Zhou et al., 2018). However, an article by Chianca and colleagues (2022) reported that *L. casei* may present virulence properties that aid biofilm formation in carious lesions. *S. mutans* and *L. casei* engage in a competitive relationship when grown together, as observed in mixed culture (Wen et al., 2010) and in the oral cavity of gnotobiotic rats (Michalek et al., 1981). The intricacies of this relationship are not fully understood, leaving a knowledge gap regarding their impact on cell cycle disruptions and the underlying molecular pathways. The nematode *C. elegans* is an excellent research model to test the effects of these bacteria because of its short life cycle, self-propagation, transparency, and the high homology between its genes and human genes. The current understanding of genes and cellular processes in *C. elegans* can help determine the specifics of the virulence factor effects.

To investigate the effects of *S. mutans* and *L. casei* on *C. elegans* , two assays were performed using *E. coli* OP50 as the control. Assay 1 tested the effects of each bacterium by growing synchronized worms on duplicate NGM plates. Assay 2 evaluated whether feeding on *L. casei* prior to transfer to *S. mutans* conferred any protective effect, using triplicate plates for each treatment. Worms were assessed over four days, and their developmental stages were examined using differential interference contrast (DIC) microscopy. Notably, worms grown on *S. mutans* exhibited delayed larval development and morphological abnormalities such as dumpy and twisted pharynx phenotypes ( Fig. 1B, 1E), and *L. casei* did not prevent such defects. To quantify these observations, statistical analyses were performed to compare developmental outcomes across treatments.

In assay 1, two independent-samples Kruskal-Wallis tests were used to evaluate whether bacterial treatments affected the number of eggs laid and the number of L4 larvae present. The test for egg counts showed a significant difference among the three treatments ( *H* = 12.47, *df* = 2, p = 0.001), with pairwise comparisons indicating a significant reduction in egg production in the *S. mutans* treatment compared to OP50 ( Fig. 1A ). The test for L4 counts ( *H* = 22.47, *df* = 2) also showed a significant overall effect, with post-hoc comparisons revealing differences between *S. mutans* and both *L. casei* and OP50 (p = 0.013 and p < 0.001, respectively; Fig. 1B ). While the fraction of eggs that hatched was lower for worms grown on *S. mutans* (see extended data), the lower number of L4 larvae was not only caused by an egg hatching defect. The eggs laid on day 0 should have all hatched by day 1. Therefore, for each plate, the number of L4 worms observed by day 3 was divided by the number of L1 plus L2 larvae observed on day 1. A Kruskal-Wallis test on these ratios again showed a significant difference between *S. mutans* and both *L. casei* and OP50 (p < 0.001 for both). These findings suggested that worms exposed to *S. mutans* produced fewer eggs and experienced delayed progression to the L4 stage compared to worms grown on OP50 or *L. casei* .

In assay 2, we evaluated whether feeding on *L. casei* prior to transfer to *S. mutans* offered any advantage compared to pre-feeding on OP50 . Each treatment had a sample size of *n* = 15 measurements. The mean number of eggs was 50 ± 18 for the *S. mutans* ( OP50 ) treatment and 51 ± 19 for the *S. mutans* ( *L. casei* ) treatment. The mean number of L4s was 12 ± 7 and 12 ± 8 for the same treatments, respectively. Two independent-samples t-tests were conducted to compare these means. The resulting p-values (p = 0.883 and p = 0.66, for eggs laid and L4 present, respectively) exceeded the 0.05 significance threshold, indicating no significant differences between the groups. Corresponding test statistics ( *t* = 0.149, *df* = 27.9 for eggs; *t* = 0.438, *df* = 27.5 for L4s) were both below 1, showing that any observed differences were smaller than the within-group variability. We also calculated the fraction of worms reaching L4 compared to the number of hatched larvae on day 1 (as described in the previous paragraph) and again found no difference (t = 0.44, *df* = 28, p = 0.66). These results suggested that prior feeding on *L. casei* did not confer a measurable benefit when worms were subsequently exposed to *S. mutans* .

Worms exposed to *S. mutans* laid fewer eggs, and some exhibited morphological defects. These defects were visible in DIC images, where some eggs from *S. mutans* -treated worms had abnormal round shapes ( Fig. 1C1 -2), in contrast to the normal oval-shaped eggs from OP50-treated worms ( Fig. 1D ). Additionally, some adult worms grown with *S. mutans* exhibited the twisted pharynx and dumpy phenotypes ( Fig. 1E ). The shorter worms were 80% as long as the control worms, and their widths varied from 80 - 160 μm, making them either small or dumpy based on the characteristics of small and dumpy worms in Cho et al., 2021. We observed a few worms grown on *S. mutans* with eggs inside that contained embryos further developed than normal, to the 2-fold or 3-fold stage, but none of the eggs had hatched. These adults with retained eggs were of a normal width, 82 ± 2 μm (n = 3) compared to 84 ± 2 μm (n = 4) for control worms, so the retained eggs did not make the worms wider or appear dumpy.&nbsp;

The effects of *S. mutans* on *C. elegans* reproduction were not unique to this bacterial strain. *C. elegans* usually responds to bacterial pathogens by increasing the expression of genes involved in detoxification and antimicrobial responses (Irazoqui et al., 2010, Tran et al., 2024). Changes in egg laying and brood size have been reported when *C. elegans* was exposed to various pathogenic bacteria or toxins (Mishra et al., 2022, Rae et al., 2010, Bashir et al., 2021, Sivamaruthi et al., 2015, Yamamuro et al., 2011). The round-egg morphology can be caused by defects in ovulation (Rose et al., 1997, Obinata et al., 2010) or a defective eggshell (Zhang et al., 2005). One result that was observed for both assays was the delay in progression through the larval stages for worms growing on *S. mutans* treatments. Similar findings have been reported for *Pseudomonas aeruginosa* CF18 and *Escherichia coli* mutants (Mirza et al., 2023, Zhang et al., 2019). These bacteria caused slowing of larval development by inducing mitochondrial dysfunction that resulted in ROS and iron imbalance (Mirza et al., 2023). For the adult phenotypes, mutations in biosynthetic pathways for actin cytoskeleton organization (Axäng et al., 2007) and collagen production (Cho et al., 2021) have been linked to the twisted pharynx and dumpy phenotypes, respectively. Future studies are required to examine subsequent worm generations to assess the long-term effects of *S. mutans* exposure and to clarify the underlying mechanisms.

In summary, *S. mutans* negatively affected *C. elegans* development regardless of prior feeding on *L. casei* . The negative effects observed after *S. mutans* exposure included reduced egg counts, egg morphological defects, and dumpy and twisted pharynx phenotypes for the adults. This project further supports using *C. elegans* as a model organism for microbial dysbiosis research by employing the oral pathogen *S. mutans* . It is the first examination of *S. mutans* in *C. elegans* , laying the groundwork for identifying molecules causing cellular aberrations that may be associated with human diseases.

## Methods

**Worm and Bacteria Maintenance**

*Escherichia coli* OP50 (from the Caenorhabditis Genetics Center) was grown at 36°C in Luria-Bertani Broth (BD #240110). *Streptococcus mutans* (ATCC 25175) and *Lacticaseibacillus casei* (ATCC 393) were grown at 36°C in Brain Heart Infusion Broth (BD #237500) and MRS Broth (BD #288130), respectively. Liquid cultures were grown weekly; agar plates were struck for single colonies every 2–3 weeks and stored at 4°C.

*C. elegans* N2 (wild-type hermaphrodite, obtained from the *Caenorhabditis* stock center, CGC) was grown on NGM plates seeded with *E. coli* OP50 at 20°C. Ten L4 worms were transferred to fresh 100 mm NGM plates every 3 days. NGM plates were made following the standard lab preparation (Stiernagle, 2006).

**Bacterial Preparation for NGM Plating**

*Streptococcus mutans* : 1 mL liquid culture was added to 99 mL BHI and grown at 36°C with shaking. After centrifugation (5000 × g, 6°C, 10 min), the pellet was resuspended in 5 mL BHI. Then, 100 µL was plated per 60 mm NGM plate. *L. casei* : 1 mL of culture was centrifuged at 13,000 rpm for 1 min in two 1.5 mL tubes. This was repeated four times until a thick pellet formed. In the final spin, 0.1 mL supernatant was left in the tube with the pellet. The pellet was resuspended, and 100 µL plated per 60 mm NGM plate.

*E. coli* OP50 was plated using the standard seeding protocol (Stiernagle, 2006). All plates were incubated overnight at room temperature.

**Assay 1**

On day 0, 30 young adults were transferred to a clean NGM plate. Then, 5 worms were placed on each 60 mm treatment plate containing bacteria (2 plates per strain) and allowed to lay eggs for 6 hours at 20°C. Adults were removed, and eggs were counted (day 0). Plates were incubated overnight. Larval stage (L1–L4), egg count, and censored (missing) worms were recorded daily for 3 days, until most control worms reached adulthood. L4 counts were based on the number of worms observed in the L4 stage or older on day 3. To calculate the fraction of hatched worms that reached the L4 stage by day 3, for each plate the number of L4 worms observed on day 3 was divided by the number of L1 plus L2 larvae observed on day 1. The censored worms only affected the L4 results, and they accounted for 1.1% of the total number of worms in this assay.

**Assay 2**

Worms were grown on OP50- or *L. casei* -NGM for ~two generations. On day 0, 15 young adults from each were cleaned on empty plates. Then 5 worms were transferred to each of six 60 mm *S. mutans* -NGM plates (3 from OP50 and 3 from *L. casei* groups). After 6 hours at 20°C, adults were removed, and eggs were counted. Days 1–3 followed the same procedure as Assay 1. L4 counts were based on the number of worms observed in the L4 stage or older on day 3. The fraction of hatched worms that reached the L4 stage by day 3 was calculated as described for Assay 1. Triplicates ensured equal treatment plate counts across all assays. Censored worms accounted for 3.2% of the worms in this assay.

**Statistical Analysis**

Independent-samples Kruskal-Wallis tests were performed using IBM SPSS Statistics to analyze the number of eggs and L4 worms and the fraction of hatched worms that reached the L4 stage quantified in assay 1. The p values reported were adjusted using the Bonferroni correction. The t-tests were run using the Art of Stat app (https://artofstat.com/web-apps) to compare the mean number of eggs and L4 worms and the fraction of hatched worms that reached the L4 stage in assay 2. Average values in the text are shown with the standard error.&nbsp;

**DIC Microscopy**

To examine and photograph the internal structures of eggs and worms, an inverted Olympus IX70 microscope was used with differential interference contrast (DIC) optics. Samples were immobilized on a 2% agarose pad stored in 10 mM levamisole in M9 buffer. M9 buffer was made following the standard lab preparation as reported in Stiernagle 2006. Using a dissecting microscope and an eyelash mounted on a toothpick (or a flattened platinum wire), individual worms or eggs were transferred from NGM plates to the pad. M9 buffer was added to surround the samples, and a coverslip was placed on top. Prepared slides were observed with DIC. Images were taken with an Olympus DP12 camera, and the contrast was improved using levels in Apple Preview 11.0. Worm dimensions were measured using Adobe Photoshop 27.0.0 and the custom measurement scale. Images were cropped, labeled and organized into panels using Adobe Illustrator 29.6.1.

## Reagents

Strains

**Table:** 

Organism	Strain	Source	Medium	Notes
*Caenorhabditis elegans*	N2 (wild-type, hermaphrodite)	CGC	NGM plates seeded with OP50	Used for all assays
*Escherichia coli*	OP50	CGC	LB Broth (BD #240110)	Standard food source for *C. elegans*
*Streptococcus mutans*	ATCC 25175	ATCC	BHI Broth (BD #237500)	Used as treatment in assays 1 and 2
*Lacticaseibacillus casei*	ATCC 393	ATCC	MRS Broth (BD #288130)	Used as treatment in assays 1 and 2

Reagents

**Table:** 

Reagent	Description	Source	Notes
Luria-Bertani (LB) Broth	Nutrient-rich medium for *E. coli* growth	BD #244620	Grown at 36°C for OP50 culture.
Brain Heart Infusion (BHI) Broth	Medium for *S. mutans* growth	BD #237500	Grown at 36°C with shaking.
MRS Broth	Medium for *L. casei* growth	BD #288130	Grown at 36°C with shaking.
NGM (Nematode Growth Medium)	Agar-based growth medium for *C. elegans*	Standard lab preparation (Stiernagle, 2006)	Maintenance and treatment plate base
Levamisole	Paralytic agent for immobilization	Sigma, 10 mM in M9 buffer	Used during DIC microscopy.
M9 Buffer	Saline buffer	Standard lab preparation (Stiernagle, 2006)	Worm handling and microscopy
Agarose	Used to create microscopy pads	Fisher, BP160, 2% in M9 buffer with levamisole	Sample preparation for DIC

## Data Availability

Description: The average number of eggs, larval stages 1-4, and censored (C) worms for each day for Assays 1 and 2 are shown with the standard errors.. Resource Type: Dataset. DOI: https://doi.org/10.22002/fpsac-8dr06

## References

[R1] Axäng C, Rauthan M, Hall DH, Pilon M (2007). The twisted pharynx phenotype in C. elegans.. BMC Dev Biol.

[R2] Bae WY, Nguyen UTT, Le TAN, Tran SH, Lee S, Hong SC, Cha KH, Choi ID, Kang K (2025). Lacticaseibacillus casei HY2782 improves the intestinal barrier and tract environment and ultimately prolongs the lifespan of Caenorhabditis elegans.. Food Funct.

[R3] Bashir A, Sun Y, Yu X, Sun X, Li L (2021). Nematicidal effects of 2-methyl-aconitate isomerase from the phytopathogen Pseudomonas syringae MB03 on the model nematode Caenorhabditis elegans.. J Invertebr Pathol.

[R4] Chang CW, Liu CY, Lee HC, Huang YH, Li LH, Chiau JC, Wang TE, Chu CH, Shih SC, Tsai TH, Chen YJ (2018). Lactobacillus casei Variety rhamnosus Probiotic Preventively Attenuates 5-Fluorouracil/Oxaliplatin-Induced Intestinal Injury in a Syngeneic Colorectal Cancer Model.. Front Microbiol.

[R5] Chianca GC, Antunes LAA, Ornellas PO, Neves FPG, Póvoa HCC, Iorio NLPP (2021). Virulence of Lactobacillus spp. misidentified as Enterococcus faecalis from children's carious dentine.. Acta Odontol Scand.

[R6] Cho JY, Choi TW, Kim SH, Ahnn J, Lee SK (2021). Morphological Characterization of
*small*
,
*dumpy*
, and
*long*
Phenotypes in
*Caenorhabditis elegans*
.. Mol Cells.

[R7] Fijan S (2014). Microorganisms with claimed probiotic properties: an overview of recent literature.. Int J Environ Res Public Health.

[R8] Han YW, Wang X (2013). Mobile microbiome: oral bacteria in extra-oral infections and inflammation.. J Dent Res.

[R9] Irazoqui JE, Troemel ER, Feinbaum RL, Luhachack LG, Cezairliyan BO, Ausubel FM (2010). Distinct pathogenesis and host responses during infection of C. elegans by P. aeruginosa and S. aureus.. PLoS Pathog.

[R10] Kang A, Eor JY, Lee J, Kwak MJ, Lee DJ, Seo E, Lee WJ, Son SH, Song M, Kim JM, Kim HW, Yang J, Oh S, Kim Y (2025). Lacticaseibacillus casei IDCC 3451 alleviates cognitive and behavioral functions by reshaping the gut microbiome and regulating intestinal barrier integrity in chronic stress animal models.. Curr Res Food Sci.

[R11] Michalek SM, Hirasawa M, Kiyono H, Ochiai K, McGhee JR (1981). Oral ecology and virulence of Lactobacillus casei and Streptococcus mutans in gnotobiotic rats.. Infect Immun.

[R12] Mirza Z, Walhout AJM, Ambros V (2023). A bacterial pathogen induces developmental slowing by high reactive oxygen species and mitochondrial dysfunction in Caenorhabditis elegans.. Cell Rep.

[R13] Mishra N, Mallick S, Negi VD (2021). Salmonella Typhimurium infection causes defects and fastening of Caenorhabditis elegans developmental stages.. Microbes Infect.

[R14] Obinata T, Ono K, Ono S (2010). Troponin I controls ovulatory contraction of non-striated actomyosin networks in the C. elegans somatic gonad.. J Cell Sci.

[R15] Rae R, Iatsenko I, Witte H, Sommer RJ (2010). A subset of naturally isolated Bacillus strains show extreme virulence to the free-living nematodes Caenorhabditis elegans and Pristionchus pacificus.. Environ Microbiol.

[R16] Rose KL, Winfrey VP, Hoffman LH, Hall DH, Furuta T, Greenstein D (1997). The POU gene ceh-18 promotes gonadal sheath cell differentiation and function required for meiotic maturation and ovulation in Caenorhabditis elegans.. Dev Biol.

[R17] Sharma AK, Dhasmana N, Dubey N, Kumar N, Gangwal A, Gupta M, Singh Y (2016). Bacterial Virulence Factors: Secreted for Survival.. Indian J Microbiol.

[R18] Sivamaruthi BS, Prasanth MI, Balamurugan K (2014). Alterations in Caenorhabditis elegans and Cronobacter sakazakii lipopolysaccharide during interaction.. Arch Microbiol.

[R19] Stiernagle T (2006). Maintenance of C. elegans.. WormBook.

[R20] Tran TD, Luallen RJ (2023). An organismal understanding of C. elegans innate immune responses, from pathogen recognition to multigenerational resistance.. Semin Cell Dev Biol.

[R21] Tsai MS, Chen YY, Chen WC, Chen MF (2022). Streptococcus mutans promotes tumor progression in oral squamous cell carcinoma.. J Cancer.

[R22] Wen ZT, Yates D, Ahn SJ, Burne RA (2010). Biofilm formation and virulence expression by Streptococcus mutans are altered when grown in dual-species model.. BMC Microbiol.

[R23] Whisner CM, Athena Aktipis C (2019). The Role of the Microbiome in Cancer Initiation and Progression: How Microbes and Cancer Cells Utilize Excess Energy and Promote One Another's Growth.. Curr Nutr Rep.

[R24] Yamamuro D, Uchida R, Takahashi Y, Masuma R, Tomoda H (2011). Screening for microbial metabolites affecting phenotype of Caenorhabditis elegans.. Biol Pharm Bull.

[R25] Zhang Y, Foster JM, Nelson LS, Ma D, Carlow CK (2005). The chitin synthase genes chs-1 and chs-2 are essential for C. elegans development and responsible for chitin deposition in the eggshell and pharynx, respectively.. Dev Biol.

[R26] Zhang Jingyan, Li Xuhang, Olmedo Maria, Holdorf Amy D., Shang Ye, Artal-Sanz Marta, Yilmaz L. Safak, Walhout Albertha J.M. (2019). A Delicate Balance between Bacterial Iron and Reactive Oxygen Species Supports Optimal C.&nbsp;elegans Development. Cell Host & Microbe.

[R27] Zhou M, Liu X, Yu H, Yin X, Nie SP, Xie MY, Chen W, Gong J (2018). Cell Signaling of Caenorhabditis elegans in Response to Enterotoxigenic Escherichia coli Infection and Lactobacillus zeae Protection.. Front Immunol.

